# Trends and future challenges in sampling the deep terrestrial biosphere

**DOI:** 10.3389/fmicb.2014.00481

**Published:** 2014-09-12

**Authors:** Michael J. Wilkins, Rebecca A. Daly, Paula J. Mouser, Ryan Trexler, Shihka Sharma, David R. Cole, Kelly C. Wrighton, Jennifer F. Biddle, Elizabeth H. Denis, Jim K. Fredrickson, Thomas L. Kieft, Tullis C. Onstott, Lee Peterson, Susan M. Pfiffner, Tommy J. Phelps, Matthew O. Schrenk

**Affiliations:** ^1^School of Earth Sciences, The Ohio State UniversityColumbus, OH, USA; ^2^Department of Microbiology, The Ohio State UniversityColumbus, OH, USA; ^3^Department of Engineering, The Ohio State UniversityColumbus, OH, USA; ^4^Department of Geology and Geography, West Virginia UniversityMorgantown, WV, USA; ^5^College of Earth, Ocean, and Environment, University of DelawareLewes, DE, USA; ^6^Department of Geosciences, Penn State University, State CollegePA, USA; ^7^Biological Sciences Division, Pacific Northwest National LaboratoryRichland, WA, USA; ^8^Department of Biology, New Mexico TechSocorro, NM, USA; ^9^Department of Geosciences, Princeton UniversityPrinceton, NJ, USA; ^10^Itasca Consulting GroupMinneapolis, MN, USA; ^11^Center for Environmental Biotechnology, University of TennesseeKnoxville, TN, USA; ^12^Department of Geological Sciences, Michigan State UniversityEast Lansing, MI, USA

**Keywords:** deep biosphere, deep subsurface, drilling, contamination, shale, deep life

## Abstract

Research in the deep terrestrial biosphere is driven by interest in novel biodiversity and metabolisms, biogeochemical cycling, and the impact of human activities on this ecosystem. As this interest continues to grow, it is important to ensure that when subsurface investigations are proposed, materials recovered from the subsurface are sampled and preserved in an appropriate manner to limit contamination and ensure preservation of accurate microbial, geochemical, and mineralogical signatures. On February 20th, 2014, a workshop on “Trends and Future Challenges in Sampling The Deep Subsurface” was coordinated in Columbus, Ohio by The Ohio State University and West Virginia University faculty, and sponsored by The Ohio State University and the Sloan Foundation’s Deep Carbon Observatory. The workshop aims were to identify and develop best practices for the collection, preservation, and analysis of terrestrial deep rock samples. This document summarizes the information shared during this workshop.

## INTRODUCTION

It has been estimated that up to 25 × 10^29^ bacterial cells are present in the terrestrial subsurface, potentially accounting for 40–60% of all bacterial cells on Earth (Whitman et al., 1998; McMahon and Parnell, 2014). The depth limit for life on Earth is unknown, but likely tied to upper temperature limits and the availability of water in terrestrial systems. Microorganisms have been detected in 3.6 km deep groundwater accessed via South African gold mines (Moser et al., 2003), in sub-sea floor sediments (Schrenk et al., 2010), and at almost 4 km beneath ice sheets in Lake Vostok (Priscu et al., 1999). However, given the extent of the deep biosphere, the majority of potential habitats remain almost completely unexplored (Edwards et al., 2012). As such, a series of wide-ranging research questions remain unanswered: what controls the subsurface microbial abundance (McMahon and Parnell, 2014)? What is the taxonomic diversity of these systems (Teske and Sorensen, 2007)? What microbial metabolisms are active across diverse chemical and physical conditions (Orsi et al., 2013)? How do cells survive exceedingly low fluxes of energy and nutrients that lead to extremely slow doubling times, and bring to question the energy requirements for cellular maintenance and repair (Hoehler and Jorgensen, 2013)? How do taxonomically similar microorganisms appear in seemingly isolated deep environments across the Earth (L’Haridon et al., 1995)? How are microorganisms impacted when human activity alters these deep subterranean and oceanic environments? These outstanding questions emphasize the importance of continued deep subsurface research, in both terrestrial and marine systems.

## SAMPLE COLLECTION AND CONTAMINATION ASSESSMENT

Recovering material from the subsurface generally requires drilling technologies to reach suitable depths, although in some instances pre-existing infrastructure may be used for sample collection (e.g., South African gold mines). A number of drilling techniques including hollow-stem auger coring, cable-tool coring, and rotary sonic are suitable for shallow sampling in unconsolidated sediments (Kieft, 2010). While these techniques can be used without drilling fluids, thus limiting potential contamination of recovered materials, they are not suitable for recovery of deeper rock and sediments. For accessing deeper materials (> 300 m), rotary drilling is generally used in conjunction with added drilling fluids. This contrasts with drilling in marine sediments, where surrounding ocean water can be used as the drilling fluid. Such fluids are frequently muds (bentonite, and other organic constituents), although formation waters, foams and gasses can be substituted in some instances. Although these fluids are essential to seal the borehole, to cool and lubricate the drill bit, and to adjust density and viscosity with the borehole, they can support extremely high densities of microorganisms, and must be carefully managed when acquiring microbiological samples (Beeman and Suflita, 1989). Preserving *in situ* geochemical and microbiological signatures during recovery of core material is technically challenging. Returning sediments to the surface from deep locations can take significant time, during which such signatures may change. Rock and sediments exhibiting high porosity and permeability may be particularly at risk to these changes. Technologies are currently being developed to design a freeze-shoe sampler that would enable the freezing of sediment and rock cores *in situ* during recovery, and thus prevent microbiological and geochemical shifts.

Samples and measurements can be obtained at multiple points during and after borehole drilling. *In situ* pore water chemistry can be estimated during drilling, via the use of devices that enable “probe-at-the-bit” measurements (Hall et al., 2008). Once a well has been developed, *U*-tube borehole fluid samplers can be used to remove the drilling fluid or monitor its dilution over time with ground water flow and then collect true formation fluids and gasses at near *in situ* conditions (Freifeld, 2009; Stotler et al., 2011). Further, geochemical conditions and microbial community structures for specific fractures can be determined through the use of packers that isolate those fractures within a borehole for sampling (Haveman et al., 1999; Shimizu et al., 2006; Purkamo et al., 2013). Finally, significant understanding of the mineralogy and geochemistry of a subsurface environment can be derived from the effective utilization of well log data (Onstott et al., 1998).

**FIGURE 1:**
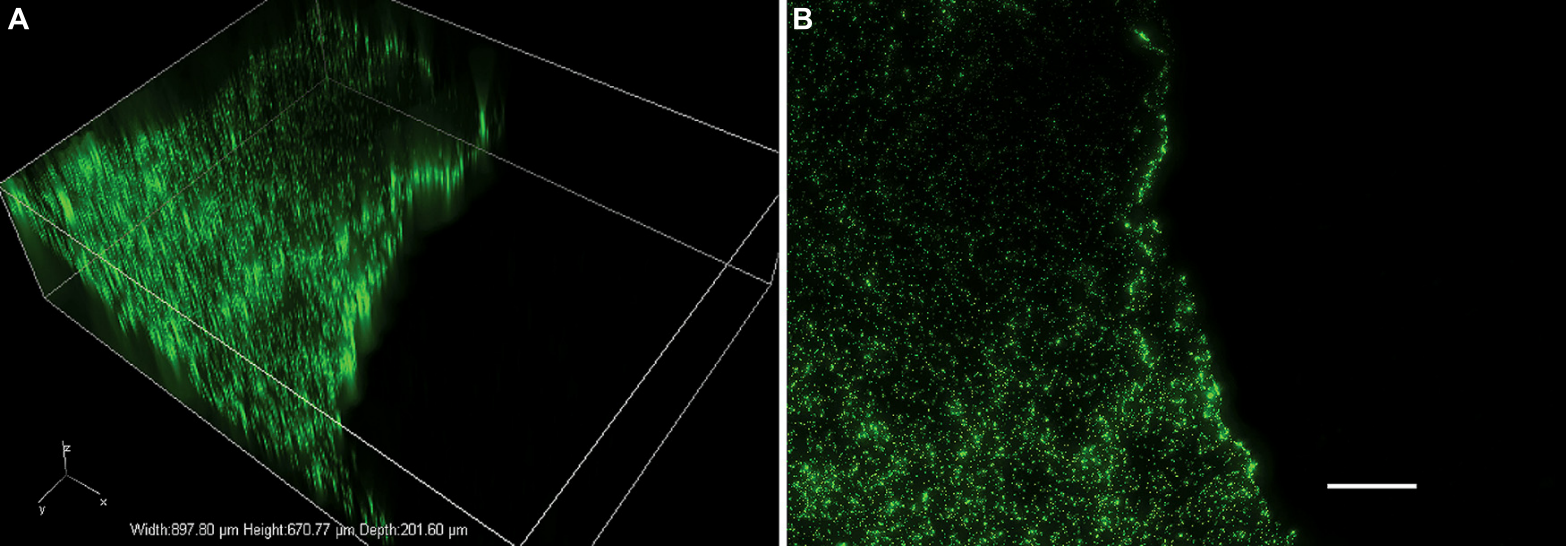
**Example of fluorescent microspheres (green dots) on shale showing contaminated and uncontaminated regions.** The 2.5 by 4 cm shale was exposed to an aqueous solution containing 0.5 μm Fluoresbrite yellow-green microspheres (Polysciences Inc., Warrington, PA, USA) at a concentration of 3.64 × 10^8^ particles/mL. Images were obtained using a Nikon Eclipse Ti inverted microscope at 100x total magnification (10x objective) and NIS Elements v. 4.00.07 software. Image **(A)** shows a volumetric composite of captured *Z*-stack images over a depth of 201.60 μm; image **(B)** shows the same data as a composite maximum-intensity projection. Scale bar = 100 μm.

When solid rock and sediment matrices are recovered, a primary concern focuses around potential contamination issues. Contamination can occur at several points during the drilling and coring process. Sources include: surface water used during drilling, air contamination of the mud tanks, additives to the drilling fluid, contaminated surfaces of the mud pumps, core barrels and drill bits, and contamination from overlying formations and groundwater via a process known as drilling drag-down. Due to the extremely low biomass in deep subsurface formations, special care must be taken to minimize microbial contamination as even a small quantity of exogenous bacteria can mask indigenous biomarkers and compromise cultivation and enrichment efforts. Common methods to assess the extent of contamination include the use of chemical, microbiological and particle tracers, with multiple, redundant, tracers recommended to ensure sample integrity (Russell et al., 1992; Kieft et al., 2007). Typical tracers include visual markers such as fluorescein and rhodamine B (Russell et al., 1992; Wandrey et al., 2010), and chemical tracers such as perfluorocarbons (McKinley and Colwell, 1996; Smith et al., 2000; House et al., 2003; Pfiffner et al., 2008; Santelli et al., 2010) and perdeuterated *n*-octacosane (*n*C_28_; Agouron Institute Drilling Project, 2014). Some of these tracers may be added throughout drilling (e.g., fluorescein), some may be changed as suites at discrete formational/depth changes (e.g., perfluorocarbons), while others are applied onto bits and core barrels prior to drilling (e.g., perdeuterated *n*-octacosane). Fluorescent microspheres 0.5– 1.0 μm diameter can be used as a proxy for bacterial cells, and quantified by microscopy (Kallmeyer et al., 2006; Kieft et al., 2007; Stroes-Gascoyne et al., 2007; Pfiffner et al., 2008; Santelli et al., 2010; Cardace et al., 2013; Yanagawa et al., 2013; **Figure 1**). Fluorescent microspheres can be added to the drilling fluid but become cost-prohibitive and impractical in the deep subsurface as large volumes of fluids are needed; instead microspheres may be deployed in the core catcher in a plastic bag that ruptures as core material enters the core barrel (Kieft et al., 2007; Pfiffner et al., 2008; Mason et al., 2010; Yanagawa et al., 2013). Microbiological tracers (e.g., active microbial cells) have also been used to assess penetration of microorganisms into core material (Zhang et al., 2005). Finally, total organic carbon (TOC) measurements in recovered material can be a surrogate for contamination from the carboxymethyl cellulose (CMC) component of drilling mud (Wandrey et al., 2010).

A rigorous assessment of contamination includes the sub-sampling of all materials coming in contact with cores before, during and after all operational steps (e.g., drilling operations, core retrieval, and sample processing). Example samples for contamination analyses include swabs from surfaces used for drilling, coring, or paring; samples of drilling muds and return cuttings, especially when new formations are encountered; samples of drill bit lubricants; and swabs/samples from core liners (Kieft et al., 2007; Pfiffner et al., 2008). Additionally, the collection of sample “blanks” at multiple points throughout drilling and sampling processing allows for the detection of environmental contaminants (e.g., air, moisture, gloves, glovebag) to distinguish from native microorganisms. Once cores have been analyzed for tracers and potential contamination has been documented, they must be immediately subsampled prior to geochemical and microbiological analyses. Common paring and disaggregation/size reduction methods utilize core extrusion (Russell et al., 1992; Kieft et al., 2007), a hammer and/or chisel, (Pfiffner et al., 2008) circular saws with hydraulic crushing, (Santelli et al., 2010) or mortar and pestle/ball mill (Herrera and Cockell, 2007) depending upon the lithology and subsequent analyses. Field samples must be immediately preserved using appropriate methods to retain competency for subsequent microbial and geochemical analyses. Where non-culturing approaches are to be used, rapid freezing is generally ideal to capture microbial community structures from molecular biomarkers (e.g., nucleic acids, proteins, and lipids). If culturing approaches are to be applied, samples should be maintained at *in situ* pressures and temperatures or refrigerated and used as soon as possible to prevent outgrowth of organisms (Haldeman et al., 1994; Brockman et al., 1998).

## MEASURING MICROBIAL BIOMASS, ACTIVITY, AND COMMUNITY STRUCTURE

A number of techniques can be leveraged to determine microbial community structure, function, biomass concentration, and activity in the deep biosphere. DNA-based analyses are tractable in these environments, and can range from single gene biomarker studies to shotgun community genomic investigations that inform microbial community structure and functional potential (Zhang et al., 2005; Chivian et al., 2008; Wrighton et al., 2012, 2013; Dong et al., 2014). Catalyzed reported deposition fluorescent *in situ* hybridization (CARD-FISH) has been used in some environments to identify active cells, while demonstrating that DNA is sufficiently intact to hybridize with primers and probes (Hoehler and Jorgensen, 2013). Recently, amino acid-based racemization rates have been used to constrain potential depth limits and temperatures for microbial activity (Onstott et al., 2013). RNA-based analyses for microbial activity are challenging in low-biomass deep terrestrial environments. Messenger RNA signatures may change during sample recovery, although the ability to sample at some deep subsurface locations (e.g., South African gold mines) may enable the rapid preservation of recovered biomass. As discussed earlier, mechanisms for freezing samples during the coring process may offer another solution to preserving signatures that would otherwise change rapidly.

Similarly, lipid biomarker profiles can be determined from intact polar lipids (PLs) or their derived fatty acid methyl esters to provide estimates of biomass and determine the relative abundance of taxonomic groups, including Eukaryotes, Bacteria, and Archaea (Fredrickson et al., 1995; White et al., 1996; White and Ringelberg, 1997; Schubotz et al., 2009). Polar lipid analyses can be used to estimate total biomass and the proportion of viable versus dead cells (Balkwill et al., 1988; Findlay et al., 1989; Fredrickson et al., 1995; White and Ringelberg, 1997). In conjunction with taxonomic identification, these analyses can be used to infer microbial phenotypic states as they relate to environmental conditions, and are therefore very useful for subsurface studies. For example, enriched trans and cyclopropyl fatty acids have been used to indicate microbial responses to stress and toxicity (Heipieper et al., 1992; White and Ringelberg, 1997), while higher proportions of cyclopropyl fatty acids versus monounsaturated and saturated fatty acids have been used to indicate microbial starvation (Guckert et al., 1986; Kieft et al., 1994). Additionally, respiratory quinones have been employed to infer environmental redox potentials (Hedrick and White, 1986; White and Ringelberg, 1997) and may be useful in environments where direct geochemical measurements are difficult to obtain. Finally, isotope signatures in PLs offer a valuable indicator of microbial function in deep subsurface environments. Isotopic compositions are determined using a gas chromatograph combustion interface isotope ratio mass spectrometer (GC-C-IRMS).

Deoxyribonuclicacid- and biomarker-based analyses require the extraction of biological material from subsurface cores, a process complicated by low biomass concentration, chemical, and physical factors (Herrera and Cockell, 2007). The nature of the matrix itself, which is often characterized by low porosity, carbonate precipitates, and brine minerals and fluids, impacts the amount and quality of extractable DNA, RNA, and lipids (Nielsen and Petersen, 2000; Herrera and Cockell, 2007; Wu et al., 2009). In many systems, cells are encased in the physical matrix and this directly affects the choice of subsampling to optimize extraction efficiency. DNA is usually extracted from environmental samples by direct cell lysis, using chemical or physical lysis, or a combination of both (Zhou et al., 1996; Griffiths et al., 2000; Hurt et al., 2001; Barton et al., 2006). Although many commercial DNA extraction kits have been developed to increase extraction reproducibility and yield, it is recommended that multiple methods be tested and compared, either on actual sample material or chemically similar samples (Barton et al., 2006; Herrera and Cockell, 2007; Novinscak and Filion, 2011; Direito et al., 2012; Paulin et al., 2013). DNA sorption onto mineral surfaces is a significant problem with low-biomass samples; although blocking agents or carrier molecules have been shown to help overcome this challenge (Barton et al., 2006; Direito et al., 2012).

Microbial activity in recovered material can be measured through laboratory batch enrichments or continuous flow-through experiments. Given that both pressure and temperature increase with depth in terrestrial subsurface environments, microorganisms living in such environments must be able to tolerate, survive, and even proliferate under these conditions. Hydrostatic pressure-adapted microorganisms are known as piezophiles, and have optimal growth rates at pressures greater than 0.1 MPa, while hyperpiezophiles require pressures > 60 MPa for optimal growth (Bartlett, 2002). Growth experiments at these pressures are therefore often desirable to obtain relevant data on cultivable organisms (Zeng et al., 2009). High-pressure devices allow recovered samples to be maintained at *in situ* pressures and temperatures. Some of these systems require no de-pressurization following core recovery, and allow pressurized material to be subsampled and incubated under *in situ* conditions (Kyo et al., 1991; Parkes et al., 2009). Other pressure core samplers have been designed to recover material from depth at *in situ* pressures (Pettigrew, 1992), and have been effectively used in the oceanic deep subsurface (Dickens et al., 2003). These samplers require de-pressurization for subsampling. *U*-tube samplers can acquire fluid and microbial samples at formation pressure and are readily adaptable to subsampling (Freifeld, 2009; Stotler et al., 2011). Although rapid de-pressurization can result in cell death, slower-rates of de-pressurization do not necessarily cause lethal damage to piezophiles (Yayanos and Dietz, 1983; Chastain and Yayanos, 1991; Park and Clark, 2002). Resulting material can be re-pressurized to desired pressures using relatively simple equipment, such as modified Hungate tubes (Bowles et al., 2011) or pressure bags (Kato et al., 2008) inside high pressure stainless steel vessels, enabling microbial batch cultivation in the laboratory. Continuous flow-through high-pressure reactors have also been developed, and recently used for determining rates of anaerobic methane oxidation (AOM; Deusner et al., 2010; Zhang et al., 2011). The ability to culture microbial assemblages under environmentally representative conditions enables rate measurements for microbially catalyzed reactions to be determined (Brockman et al., 1998; Deusner et al., 2010; Tamegai et al., 2012), while enrichment of specific microbial groups can be used to obtain either pure cultures or enriched microbial consortia. Resulting biomass provides abundant material for omics-based analyses of functional potential, such as transcript expression or proteomics. In addition, cultivation at high pressures and temperatures can be used to remove contaminant species that are unlikely to tolerate such extreme conditions.

## PHYSICAL AND CHEMICAL CHARACTERIZATION OF ROCK CORE AND FLUIDS

In recovered rock samples, the development of linkages between pore structure and microbial parameters is key for understanding the distribution of microbial communities. In fine-grained shale systems, source rocks have low porosities and extremely low permeabilities, on the order of nanodarcies (Javadpour, 2009; Sondergeld et al., 2010). Understanding the microstructural controls on porosity and permeability has implications for the nature of biodiversity in such systems, in that it governs the movement of cells and chemicals within the rock. Conversely, other host rocks such as sandstone and carbonate systems can have higher porosities and permeabilities, with greater potential for microbial and chemical transport through fractures and matrix pores in such formations (Fredrickson et al., 1997; Dong et al., 2014). Currently, a range of new imaging technologies can be used in concert with more conventional characterization methods (Bryndzia and Braunsdorf, 2014). The advent of focused ion beam – scanning electron microscopic (FIB – SEM) techniques now allows us to image pore networks in the rock matrix (Curtis et al., 2012). This 3-dimensional method is part of a broader suite of instruments that image rock samples including X-ray and neutron computed tomography – XCT and NCT (Perfect et al., 2014). Recently, the application of Small and ultra-small angle neutron scattering (USANS) has proved a valuable complement for the analysis of porosity and pore connectivity at the nanometer to the centimeter scale (Anovitz et al., 2013; Jin et al., 2013). Sample preparation for X-ray and neutron tomography of native rock core only requires the material to be sized (length and diameter) according to the desired resolution of the instrument – e.g., imaging the pore features < 1 micron requires samples ranging from a few to 10’s of mm^3^. Neutron scattering is conducted on ~150 micron-thick polished sections pressure impregnated with epoxy and mounted on 1 × 2 inch quartz slides (Anovitz et al., 2013). For higher resolution assessment down to the nanoscale by dual beam-FIB, a small chip or core roughly a few mm’s on a side or diameter, respectively, is used (Curtis et al., 2012).

Given the highly heterogeneous nature of pore networks and fractures in many rock types and samples, determining spatial aspects of microbial activity is important, yet technically difficult. In a novel experimental setup, microautoradiography techniques were applied to core material to determine spatial locations for microbial sulfate reduction. *In situ* ^35^S-sulfate reduction was monitored using freshly fractured cores wrapped in silver foil (Russell et al., 1992; Fredrickson et al., 1997; Krumholz et al., 1997). ^35^S-sulfide was retained on the foil and offered a two-dimensional image of discrete pockets of microbial activity that could be mapped to physical and chemical characterizations of the core. These activity measurements can be directly related to cation and anion analyses of pore waters trapped in cores using crush and leach methods and correcting for drilling fluid contamination using tracers. Stable N-isotope analyses have become sensitive enough to obtain the N and O isotopic compositions of pore water trapped in rock with only 1% porosity (Silver et al., 2012). Formation gas composition and its isotopic signatures provide valuable information on whether methanogenesis is taking place within the formation and is typically monitored during drilling. The pore water gas compositions of cores can be measured by quickly transferring intact cores into evacuated leak-tight cylinders. The cores then degas into the cylinders and are sampled for gas composition and even for noble gas dating of the pore water (Lippmann-Pipke et al., 2011).

Other chemical signatures in rock matrices can be analyzed using isotope analysis tools to infer carbon pools, or determine potential microbial activity. The carbon isotopic composition of TOC is an excellent indicator for determining the source and type of organic matter, given that diagenetic alterations or removal of organic matter pools does not significantly affect the δ^13^C of bulk organic matter in sedimentary rocks, particularly in black shales (Meyers, 1994; Bekker et al., 2008; Young et al., 2008; Ader et al., 2009; Jiang et al., 2012). Conversely, N and S isotope fractionations in such media are closely associated with microbial processes like sulfate reduction and denitrification (Berner, 1978; Gautier, 1986; Beier and Hayes, 1989; Altabet, 2006; Quan et al., 2008). The bulk δ^13^C and δ^34^S also serve as excellent tracers for microbial oxidation of methane via sulfate reduction as this process leaves the sulfate pool enriched in ^34^S while adding light ^12^C to the total carbon pool (Kemp and Thode, 1968; Jahnke et al., 1995; Freeman, 2001; Seal, 2006). The C, N, and S isotopes are conservative and do not get altered by exposure of the core to air and mild temperature changes during storage. However, as with previously described analysis techniques, microbial contamination from drilling fluids and muds and their potential to impact paleoenvironmental signals is best avoided by using material from inner portions of recovered cores.

## FUTURE CHALLENGES

Despite advances in deep biosphere sampling techniques, and development of high-resolution molecular analysis tools, a range of challenges still exists in understanding these environments. How representative are collected samples? How can samples be better preserved for downstream analyses? Advances are currently being made in sample collection efforts, with development of freeze-shoe samplers that can freeze sediment cores *in situ*. Perhaps the greatest challenge is developing a predictive understanding of microbial processes in such environments based on a limited number of expensive, difficult to collect vertical borehole samples. Characterization will require greater linkages between biogeochemical, geophysical, mineralogical, and microbiological data, and the presentation of these results in a regional and global context. Additional research is also needed to determine the effects of engineered activities (e.g., hydraulic fracturing, geologic CO_2_ sequestration) on shale and other rock-hosted biodiversity, as the scale of these activities has the potential to promote significant change within the terrestrial subsurface. Using best practices for the collection, preservation, and analysis of biological and chemical signatures of these samples is key to advancing our understanding of the deep biosphere.

## WORKSHOP PARTICIPANTS

David R. Cole (Coorganizer), Paula J. Mouser (Coorganizer), Shihka Sharma (Coorganizer), Michael J. Wilkins (Coorganizer), Kelly C. Wrighton (Coorganizer), Jennifer F. Biddle, Elizabeth H. Denis, Jim K. Fredrickson, Thomas L. Kieft, Tullis C. Onstott, Lee Peterson, Susan M. Pfiffner, Tommy J. Phelps, Matthew O. Schrenk.

## Conflict of Interest Statement

The authors declare that the research was conducted in the absence of any commercial or financial relationships that could be construed as a potential conflict of interest.
